# Safety and Quality of Milk and Milk Products in Senegal—A Review

**DOI:** 10.3390/foods11213479

**Published:** 2022-11-02

**Authors:** Cortney Leone, Harshavardhan Thippareddi, Cheikh Ndiaye, Ibrahima Niang, Younoussa Diallo, Manpreet Singh

**Affiliations:** 1 Department of Poultry Science, University of Georgia, Athens, GA 30602, USA; 2 Cereal Science Department, Institut de Technologie Alimentaire (ITA), Dakar 2765, Senegal; 3 Department of Food Science and Technology, University of Georgia, Athens, GA 30602, USA

**Keywords:** dairy, milk, food safety, quality, Senegal

## Abstract

Historically, local milk production in Senegal has struggled to keep up with the demands of consumers, so there has been a heavy reliance on imported milk and milk products. More recently, efforts have been made to improve local dairy production by establishing large, organized dairies that collect milk from rural production areas and developing small-scale processing units, such as mini dairies. The local dairy value chain in Senegal consists of (1) informal collection systems where farmers commonly deliver milk directly to dairies; (2) traditional and artisanal processing using simple equipment and techniques; and (3) short local marketing and sale circuits. Most West African dairy sectors are dominated by raw, unpasteurized milk or traditional, spontaneously fermented milk products, such as *lait caillé* in Senegal, sold through small-scale channels without a cold chain, so the risk of food safety hazards may be increased. Microbiological, chemical, and physical hazards have been found in milk and milk products across West Africa. There is a need to educate milk producers, small-scale processors, and vendors on the importance of refrigerating milk immediately after milking as well as maintaining the cold chain until the milk is heat treated and, subsequently, until the milk is marketed to the consumer. However, without assistance, obtaining the equipment necessary for cold storage and processing of milk can be challenging.

## 1. Introduction

Food safety can affect trade, market access, productivity, human health, livelihood, and, ultimately, can be a cornerstone for economic growth. The literature clearly shows the connection between food safety, health, nutrition, food security, market access, trade, socio-economic impact, gender, and youth. With the help of public health campaigns, consumer awareness of food safety is increasing, and African consumers are beginning to prefer assurances of safe foods particularly in urban and more affluent areas [1].

**Figure 1:**
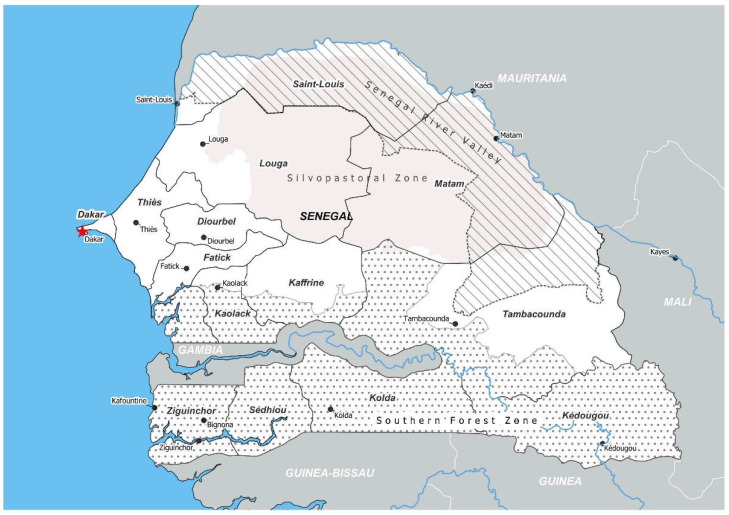
Map of the Republic of Senegal including major cities, regions (outlined in solid black), and agroecological zones (patterned areas).

The Republic of Senegal is a West African country with a population of 16.7 million people, and 25% of the population lives in the area around the capital city of Dakar [2] (Figure 1). Senegal is characterized by two main seasons including a rainy season from June to October with hot, humid monsoons and a dry season with cool, dry winds from the north. The southern part of Senegal has a wet climate with rainfall of more than 1000 mm per year while the northern regions receive less than half that amount [3]. Senegal’s economy is largely based on the agricultural sector with 29.5% of households practicing livestock farming, including for milk production. Livestock and milk production contribute to food and nutritional security for many households, and there have been efforts by the government to enhance milk production in different regions [4].

Senegal faces significant challenges in its efforts to improve food safety, such as a wide range of market types (formal and informal, domestic and export), changing food production and processing systems, underdeveloped food safety infrastructure, and complex and fragmented regulatory and governance systems for food safety [5]. Food safety is a significant public health issue, and the World Health Organization (WHO) conservatively estimates that Africans suffer 127 million acute illnesses and 91,000 deaths annually from foodborne hazards with the largest burden of disease falling on children below the age of 5. In addition, Sub-Saharan Africa (SSA) has the highest per capita incidence of foodborne illness in the world [1]. Commercial purchasers and consumers alike are increasingly expecting safe products and may react negatively, to the detriment of market access, when their expectations are not met.

In 2017, national milk production in Senegal was estimated at 243.5 million liters with more than half coming from pastoral livestock. In the same year, imported milk and dairy products reached 211.6 million liters and consisted mainly of milk powder (93%). A large amount of imported milk products indicates great potential for local production and processing of milk [6]. The development of intensive and semi-intensive dairy systems has led to an increase in milk production in Senegal. For example, a 66% increase in local milk production was observed from 2008 to 2018 [7]. In addition, the organization of the dairy value chain from production, processing, and distribution is needed and can enhance economic development in rural areas through the establishment of small- and medium-scale entrepreneurs (SMEs) and the replacement of imported products with local products. The development of the milk trade in Senegal has led to the emergence of artisanal processing enterprises. 

Milk is produced by individual farmers in Senegal and aggregated prior to transportation to urban centers. The lack of a cold chain results in the significant deterioration of microbiological quality and the potential growth of foodborne pathogens. Milk is processed into a variety of traditional milk products by small-scale processing units or processors, and the final products include naturally fermented yoghurt-like milk produced with slight variations in processing methods. Small-scale processors of traditional milk products often lack pasteurization, storage, and packaging facilities and do not adhere to Good Manufacturing Practices (GMPs) or Good Hygiene Practices (GHPs). The processing of milk into yoghurt-like fermented milk and other fermented products relies heavily on back-slopping where a portion of a previous batch of product is used to start fermentation in a new batch. A lack of adherence to GMPs, including uncontrolled fermentation due to a reliance on back-slopping in the traditional fermentation processes, may render the milk, and thus the fermented products, susceptible to contamination with human pathogens of public health concern. The main issues along the dairy value chain include the shelf life and microbiological safety of processed dairy and dairy products.

Modern processing technologies have the capacity to improve the nutritional status of vulnerable populations, such as those suffering from malnutrition or nutrient deficiencies, by generating high-quality, market-competitive, value-added products to expand local and regional markets. Yet significant gaps remain in the application of these technologies due to the lack of implementation of regulatory policies for safe food manufacturing.

## 2. Overview of the Dairy Value Chain

Local milk production in Senegal is low and only covers 55% of the demands of the population (30.2 L per capita) [6]. To make up the difference, there is a heavy reliance on imported milk and milk products [8,9]. The dairy sector comprises two main parts: (1) imported dairy products, especially powdered milk, and (2) local dairy production and processing from traditional agro-pastoral communities [10]. Efforts to reduce imports and improve local production of dairy in the recent past have had little success [8,11]. According to Boimah and Weible [12], the estimated consumption of milk is continuously increasing in Senegal. However, the increase is supported by imported products from European countries and not domestic milk production. The packaging and distribution of imported milk powders by Senegalese companies do not facilitate the promotion of local milk production and marketing. Instead, the establishment of large organized dairies, such as La Laiterie du Berger, that collect milk from rural production areas and the development of mini dairies in milk production zones could be promising endeavors to increase national production.

The continued growth of the food processing industry in Senegal could facilitate a change in this pattern of reliance on imported milk as well as valorize milk and milk products by investing in more mini dairies and enhancing the national collection of milk [13]. Consumers in Senegal have a strong preference for local and domestically produced and processed milk in terms of quality attributes compared to imported milk. However, imported milk and milk products are more accessible and diverse which influences the consumer’s decisions when purchasing [12]. More work on improving the diversification and quality of milk products is needed. Here, we describe the components of the local dairy value chain in Senegal and how it plays a role in providing opportunities to enhance local and domestically processed products.

### 2.1. Production

Three main types of dairy production exist in the North and North Central regions of Senegal corresponding to Senegal River valley and silvopastoral zones, respectively (Figure 1). First, the extensive or traditional production system is characterized by transhumance during the dry season. In this system, 38% of the national milk supply is produced within the regions of Saint-Louis, Matam, and Louga for consumption and any surplus is sold in the local marketplace [14]. Second, the semi-intensive system is an enhancement of the traditional system with regard to the management of animals and the organization of production where the main concern becomes continuous milk production in all seasons. In this system, the milk produced by nearly 25% of the cattle is considered a secondary source of income, rather than a source for self-consumption, for related actors in the North. Furthermore, the decrease in natural resources which requires the use of supplements could explain the higher production costs in this zone [8,15]. The Kolda, Ziguinchor, and Tambacounda regions belonging to the South, where nearly 45% of the cattle population is registered, are important semi-intensive livestock production areas with high rainfall producing abundant natural vegetation for more potential milk production. In these zones, crop residues (peanut and rice fodders, cottonseed, and sesame cake) are fed to the animals, and low land pressure allows for large pastures. The combination of natural resources with the intensification of animal husbandry leads to reduced production costs [16]. Third, the intensive production system, which is mainly found in peri-urban areas, uses exotic breeds of cattle for milk production rather than local breeds. The production levels of this system are much higher than the other systems because of the high level of inputs needed especially feed, the use of biotechnology to improve production, and the use of specialized hired labor [8,9,10,17].

### 2.2. Collection

There are three types of milk collection systems in Senegal: (1) farmers deliver milk directly to the dairy, (2) private collectors of the dairy gather milk from nearby rural communities, and (3) farmers bring milk to a collection center where the dairy acquires the milk. The first system is the most common, and collection centers are rare [10]. Milk collection in Senegal is almost entirely informal. For example, Bankole et al. [18] revealed that in the Kolda region, most milk collectors surveyed were professionals who transported milk mainly by bicycle and most did not treat the milk collected, but instead delivered it to processing units (62%), vendors (29%), or directly to consumers (5%). More than 80% of milk is collected and processed from farms of traditional Fulani women following these informal channels [19]. An improved collection channel is developing in production areas where technical supervision and support is received on production and processing equipment through funded projects or programs. Additionally, a modern channel initiated by the private company La Laiterie du Berger has been introduced. This large organized dairy is the only dairy of its kind that uses milk collected solely from producers in Senegal rather than relying on imported or powdered milk. It uses a 40 to 50 km radius network for collecting fresh milk using mobile centers (motorcycles) and storage systems (cans). This channel is facing difficulties related to seasonal disparities in milk collection due to practices of transhumance in the dry season, costs for transferring milk to the factory, and often poor microbiological quality. To alleviate this problem, La Laiterie du Berger assists milk producers by establishing forage areas, modern collection centers, etc., to facilitate milk production during the lean season.

### 2.3. Processing

In 2021, Senegal had more than 500 dairy-processing units that could be grouped according to their production capacity and type of technology into artisanal units, mini dairies, semi-industrial units, and industrial units. Traditional and artisanal processing using simple equipment and techniques makes up the majority of the processing units which are often located in secondary towns such as Saint-Louis, Dahra, Tambacounda, Velingara, and Kolda [8,10]. Most traditional processing relies on spontaneous fermentation with or without pasteurization of the milk prior to fermentation, and the main product is *lait caillé*, or curdled milk [10,20,21].

Since the late 1990s, the development of small-scale processing units, or mini dairies, has aimed to improve the production of local milk. Mini dairies can be either individual private enterprises or collective bodies where owners or managers handle the technical and financial operations as well as relations with suppliers and vendors. The quantities of milk collected by mini dairies have increased yearly along with increasing numbers of active units [11]. One reason for this could be that the farmers who supply mini dairies benefit from a secure and regular source of income [10]. Along with increased milk collection, mini dairies aim to increase processing margins by reducing processing costs and diversifying the types of products sold as well as guaranteeing the outflow of products [11]. In 2005, the “Guide to Good Hygiene Practices: Quality Control in Dairy Processing in Senegal” was implemented and was updated in 2011. It was designed for small artisanal and semi-industrial businesses and describes recommended hygiene practices for the production and sale of safe dairy products [10,16]. Finally, modern milk processing is performed at some semi-industrial and industrial units and generally uses powdered milk or a blend of local raw milk and powdered milk [9].

### 2.4. Marketing and Sale

The main actors in the marketing and sale of milk and milk products include (1) individual hawkers or vendors, (2) dairy cooperatives, (3) wholesalers, and (4) retailers. In the traditional extensive system, marketing and sale have a short circuit. Livestock owners often consume a part of the milk, and the rest is either sold fresh or processed before being sold at the farm, village, town, or rural roadside. Milk can be marketed at kiosks and markets or directly door-to-door to consumers. The marketing of milk by individual sellers is more important during the rainy season when production is high [8,9,10].

In high-production areas, fresh milk is sold directly to wholesalers, dairy cooperatives, and collectors for processing by artisanal and/or modern dairies. This network also uses short marketing and sale circuits. Dairy cooperatives help facilitate the collection and sale of milk around their milk collection centers. Wholesalers buy milk in bulk from either producers or collectors and sell to retailers that typically operate small holder shops in markets and mobile vending facilities. However, local milk production is not adequate to meet consumer demands, so wholesalers import milk and milk products that they then sell in wholesale or retail which uses long marketing and sale circuits [9,10].

### 2.5. Dairy Value Chain

Over the last few decades, Senegal has initiated an organized dairy value chain with involvement from research associations, extension services, and national institutions. There is a solid working relationship between (1) producers and/or dairy cooperatives and milk collection centers, (2) milk collectors and some milk processing units, (3) milk processing units and retail sellers, (4) importers and exporters of milk and milk products, and (5) retail sellers and consumers. However, a number of limitations impede the further development of the dairy value chain. Some of these include seasonality of production, poor access to production sites, poor production by local cattle breeds, poor milk hygiene practices, poor milk collection systems, and, more importantly, lack of the indispensable cold chain [9,11,18].

## 3. Overview of Milk and Milk Products

Milk is the most commonly consumed source of animal protein in Africa [22]. While a large majority of the dairy consumed in West Africa comes from imported milk and milk products, there are a number of products produced using local milk processed by traditional methods [9]. *Warankasi* or *waragashi* is a type of fresh cheese produced and consumed by Fulani and Peuhl groups in Benin, Nigeria, and northern Togo [23,24,25]. It is based on the coagulation of cow’s or goat’s milk by the calotropin enzyme which comes from the stems or leaves of the *Calotropis procera* plant, also known as the silk tree or apple of Sodom plant [23,24,26]. After coagulation, the curd is cooked, drained, and molded before either being sold or being further processed by storing in whey, treating with sorghum panicle (*Sorghum vulgaris*) or young leaves of teak (*Tectona grandis*) for coloring, exposing to sunlight, smoking over a wood fire, or frying in oil [23,24,27].

Two types of ripened cheeses are produced in West Africa following traditional processes: *touaregh* from Mali and *tchoukou* from Niger. *Touaregh* is prepared from fresh sheep, goat, or cow’s milk and rennet. These are stirred using a stick soaked in the rennet. The coagulum is dried on a mat, and the cheese is dried in thin pieces on tree branches [25]. *Tchoukou* is made from cow’s or goat’s milk or a mixture of both with rennet coagulation. After coagulation, the curd is dried on a mat for 24–48 h, depending on the time of year [25,27,28,29].

A liquid butter product produced in Niger is called *nebam* in Fulani, *man chanu* or *doungoulé* in Hausa, and *ghee* in Zarma. This product is made by heating solid butter until a certain quantity of water evaporates. *Nebam* is stored in gourds containing curds or whey [27].

Fermented dairy products, such as yogurt or sour milk, are very common in Africa due to their high nutritional value and longer shelf life [27]. In Burkina Faso, the Fulani community uses calabashes, gourds, or clay pots seeded with a natural microbial inoculum for fermentation. The containers are filled with fresh milk, covered, and placed indoors. The milk coagulates, and the whey and proteins are homogenized [30]. In Ghana, the Fulani community produces *nyarmie* using spontaneous fermentation of cow’s milk without starter cultures. The milk is sieved, pasteurized, cooled, and the fat that has accumulated on the surface is collected. The product is partially covered and left to sit overnight resulting in the formation of curdled milk. The curdled milk is stirred vigorously or whipped with a wooden stirrer to give a slightly smooth product with some suspended curds [31]. *Nunu* from Ghana and *nono* from Nigeria are spontaneously fermented milk products produced by the Fulani and Hausa communities. *Nunu* is prepared from raw cow’s milk kept in calabashes, gourds, clay pots, or rubber containers and is left to ferment for 24 h. The product is churned and some whey and butter is removed leaving a thick, yogurt-like product [27,32].

In Senegal, the dairy market consists mostly of *lait caillé*, a naturally fermented milk product [10]. In the traditional preparation of *lait caillé*, cow’s milk is filtered and heated in an aluminum pot until almost boiling. The milk is cooled, transferred into a wooden bowl or *lahal* and topped with a straw cover. The milk is left to ferment for 12–24 h depending on the season. The fat is removed, and the fermented product is homogenized using a wooden stick called a *burgal* [21]. The same *lahals* are used repeatedly, so the biofilm created from previous fermentation cycles becomes the starter for the fermentation of the next batch [20]. The bacterial community of *lait caillé* contains a wide variety of genera but is dominated by *Streptococcus* and *Lactobacillus* followed by *Lactococcus* and *Acetobacter*. There is also considerable product-to-product variation in the bacterial communities because of the uncontrolled nature of the spontaneous fermentation process used [20,21]. *Lait caillé* is commonly sold in secondary towns and rural markets as well as in small quantities for a high price in Dakar [10].

There are also a variety of new products manufactured locally in Senegal that target a wider clientele than traditional products. These include *lait caillé* and liquid yogurt products made from powdered milk instead of fresh animal milk sold at boutique shops, self-service, and mini-markets. They can also be sold in micro-doses or frozen [10,21]. Small and medium companies that transform reconstituted powdered milk have also started offering new products such as yogurt in jars, *petit-suisse*, ice cream, and reconstituted milk pasteurized in bottles or sterilized in bricks. Other products made from local milk include goat cheese and, more rarely, cow’s milk cheese which can be sold in supermarkets and mini-markets in the cities or tourist areas or sold directly to hotels and restaurants [10].

In 2006, the modern dairy processing operation La Laiterie du Berger was started. The company’s mission was to build a stronger dairy sector in Senegal with more productive livestock that are able to supply the local market. La Laiterie du Berger is the third largest producer of fresh milk dairy products in Senegal but the only one to use locally sourced milk. They use milk collected from 800 local farms to produce commercial dairy products under the Dolima brand, which means “give me more” in Wolof. La Laiterie du Berger’s network collects fresh milk twice a day from farmers within a 50 km radius of the factory in the Richard Toll area of Northern Senegal. The team also assists breeders with veterinary care and the supply of animal feed during the “lean” period when grasses become scarce. Dolima products produced by La Laiterie du Berger include yogurts, pasteurized fresh milk, crème fraîche, *thiakry* (a sweet, creamy, and mildly tangy dessert-like rice pudding or tapioca pudding), and *doolé* (a fortified *thiakry* for school children) which are available in shops, convenience stores, and supermarkets in Dakar and regional towns [33].

## 4. Food Safety Hazards in the Dairy Value Chain

Milk is expected to be free from contaminants at the point of milking and therefore safe for human consumption. However, the introduction of pathogenic microorganisms can occur at multiple stages in the dairy value chain from production to consumption [34,35,36]. Factors that can affect the safety of milk include the health of the cows, hygiene during milking and pre-storage conditions, storage conditions, farm management practices, geographical location, and season [37,38]. Hygiene measures should be implemented along the dairy value chain in order to minimize the food safety risks of milk and milk products [36,37]. Most West African countries have an informal dairy sector dominated by raw, unpasteurized milk or traditional, spontaneously fermented milk products sold through small-scale channels without a cold chain, so the risk of food safety hazards may be increased [18,35,39]. Three main categories of food safety hazards affect the quality of milk: microbiological, chemical, and physical, and a brief description of each is provided below.

### 4.1. Microbiological Hazards

Fresh milk straight out of the udder of a healthy animal is expected to be free of pathogens, but this is rarely the case. Pathogenic microorganisms can be introduced into milk in two ways: (1) endogenous contamination which occurs when pathogens are transferred directly from the blood of an infected animal into milk or from an infection of the udder and (2) exogenous contamination which occurs when milk is contaminated either during collection by the exterior of the udder, collection equipment, or hands of the collector or after collection by animals, the environment, or handlers [40]. The high nutrient content of milk provides an ideal environment for the rapid growth of microorganisms once contamination occurs [41].

Microbiological hazards in dairy products can include pathogenic bacteria, yeasts, viruses, and/or parasites. Bacterial contaminants that no longer affect milk quality in most developed countries but may still remain a problem in dairy products of developing countries include *Brucella abortus, Coxiella burnetti*, and *Mycobacterium bovis* [39,42,43]. Other pathogens found in milk and milk products across West Africa include *Bacillus* spp., *Escherichia coli*, *Listeria monocytogenes, Salmonella* spp., *Staphylococcus aureus*, and *Yersinia enterocolitica* [42,44,45]. Contamination by fungi, especially yeasts, can also occur in milk. Pathogenic yeast species in the *Candida* and *Yarrowia* genera occur most often in West Africa [46,47]. While the presence of viral pathogens in West African milk has not been investigated, milkborne viruses could include astrovirus, central European encephalitis virus, coronavirus, enteroviruses, hepatitis A (HAV) and E viruses (HEV), norovirus, and rotavirus. The parasite *Toxoplasma gondii* is the most common parasite found in milk, but like viruses, literature on the presence of this parasite in West African milk is lacking [39].

#### 4.1.1. Production and Collection

Contamination of milk generally occurs during or after milking by microorganisms from the farm environment, the interior of the teat, or the surfaces of the milking equipment. In the farm environment, soil, feed, bedding, and feces can adhere to the exterior surface of the udder and contaminate milk during collection [48]. Dairy animals in West Africa have a higher risk of ingesting contaminated feed and water because of the reliance on small-scale traditional production systems where animals are fed on grass or crop residues or are left to roam the land and graze [36,49]. After ingestion, surviving microorganisms, especially spore-forming bacteria such as *Clostridium* or *Bacillus*, can be shed into the farm environment where they can subsequently contaminate the teats and udders of dairy animals [48].

Microorganisms on the udder can enter the teat canal and cause an infection or inflammation resulting in mastitis. Mastitis may be classified as clinical, showing recognizable symptoms, or sub-clinical, showing no apparent symptoms. Sub-clinical mastitis may be more of a threat because the lack of symptoms makes it hard to recognize suffering animals [48]. In Africa, prevalence rates of clinical and sub-clinical mastitis of 4.8–26.5% and 16.3–85.3%, respectively, have been reported [50,51,52]. However, focus groups conducted in Senegal revealed that most participants did not believe that animals could carry diseases that also affect humans [35]. This lack of awareness could contribute to the consumption or sale of contaminated milk.

Finally, contamination can occur during milking. Practices, such as milking with unclean bare hands, not cleaning the teats before milking, and use of non-sanitized collection vessels, can cause microbial contamination of milk. Most small-scale farms in West Africa do not have strict procedures for cleaning and disinfecting milking containers [36]. A study from Burkina Faso found that only 9 out of 22 farms cleaned udders before milking but only if there were feces on the udders. Even after cleaning, calves were allowed to suckle to stimulate let down before milking began. Additionally, farmers on 8 of 22 farms dipped their fingers in the bucket during milking with no obvious handwashing occurring prior to milking, and two farmers dipped the teats in the bucket during milking [49]. Similarly, a study from Mali showed that handwashing before milking was rare, and the person milking would soak his hands in the already collected milk to lubricate the teat [53]. A study conducted in Northern Côte d’Ivoire also found a lack of handwashing and cleaning of udders before milking, as well as the storage of milk in unsuitable containers, such as those previously used for oil or chemicals and those that have not been properly cleaned [54]. Millogo et al. [49] reported that milk was collected in plastic buckets or calabashes in peri-urban areas of Burkina Faso. In Southern Senegal, milk is collected in plastic containers that are cleaned by either using hot water only (13%), water at ambient temperature with detergent (56%), or hot water and detergent (31%) [18].

#### 4.1.2. Storage and Transport

Safe storage after collection and during transport is essential to assure good microbiological quality milk. Strict time and temperature controls are critical to prevent the growth of foodborne pathogens in freshly collected milk prior to processing. In the United States, raw milk is required to be cooled to 7 °C or below within 6 h to prevent microbial growth before processing [55]. In the European Union, raw milk must be cooled to 8 °C if collected daily or 6 °C if not collected daily [56]. However, most small-scale operations in West Africa lack modern cooling facilities, such as refrigerators or cooling tanks, due to the high cost of the initial investment, the operational costs, and the lack of or unreliable supply of electricity [36]. Millogo et al. [49] reported that 21 out of 22 farmers had no cooling system in Burkina Faso, and 95.2% of participants never cooled milk after collection in Senegal [35]. In Northern Côte d’Ivoire, milk was stored for more than two hours after collection at ambient temperatures of approximately 30 °C [54].

Additionally, farms that produce milk in West Africa may be in rural areas far from urban markets with poor road networks or a lack of public transportation. In the Kolda region of Senegal, transport of milk by collectors took less than 1 h for 43.6% of surveyed participants, 1 to 2 h for 23.1% of participants, and 2 to 4 h for 33.3% of participants. Of these, the majority (94.8%) used bicycles to transport the milk while 2.6% each transported milk by foot or public transportation. The same study found that transport times in the Western Region in The Gambia were 27.3%, 18.2%, and 54.5% for less than 1 h, 1 to 2 h, and 2 to 4 h, respectively, and transportation was generally done by public transport (77.2%) followed by bicycles (18.2%), and foot (4.6%) [18]. In Burkina Faso, 13 of 22 farms used a bicycle to transport milk, and transportation times were 1 to 2 h. Nine farmers used a motorcycle or car to transport milk which decreased the transportation time to about one hour [49]. Similarly, Sanhoun et al. [54] found that milk in Northern Côte d’Ivoire was transported at an ambient temperature for more than three hours.

#### 4.1.3. Processing

Most of the raw milk in West Africa is either consumed directly or transformed into traditional fermented dairy products at home and is rarely heat treated or boiled [18,35]. When milk is collected at small-scale collection centers, it may be chilled or heat-treated prior to the conversion to fermented products. However, even in small-scale milk processing facilities, GMPs and GHPs, as well as proper cleaning and sanitation of equipment, are often not followed. The lack of these practices leads to a poor microbiological quality of milk and potentially failed fermentation, leading to varying microbiological quality of the final fermented products [34,36].

Production of traditional dairy products is driven by cultural practices and beliefs. Many small-scale processors do not have formal training and rely on learning by seeing, hearing, and practicing recipes that have been handed down from generation to generation [35,36]. Some farmers also do not believe in boiling milk as they believe it causes mastitis, results in udders drying up, and leads to the death of dairy cattle [35]. Often, they hesitate to sell milk to other processors who may heat treat the milk such as mini dairies [54]. The majority of participants surveyed in Senegal indicated that they never boiled raw milk before consumption (92.8%) or prior to fermentation (96.7%) at home [35]. Similar observations were made in Côte d’Ivoire and other West African countries [57]. In addition, small-scale processors, such as mini dairies, may lack the proper equipment for pasteurizing the milk. Even if they do possess the equipment, they do not have a consistent supply of electricity or cannot afford electricity due to high prices [36].

The microbiological quality of fermented milk products varies due to the practice of utilizing raw milk or the back-slopping method and improper temperatures and inconsistent times for fermentation. In Senegal, one study found that 37.9% of dairy farmers and milk processors regularly produced spontaneously fermented curdled milk at home while 96% never used lactic acid bacteria and/or enzymes to produce fermented milk at home [35]. Fermentation practices are often handed down through generations, and it is rare that modern fermentation systems utilizing good quality milk, pasteurization, starter cultures, and proper fermentation temperatures and times are followed resulting in the poor and inconsistent microbiological quality of the final products [34,58].

#### 4.1.4. Vendors

The introduction of food safety hazards and an increase in their risk can occur during the transportation of milk to vendors or during the storage and marketing of the milk to consumers. Vendors in West Africa often transport milk by foot or other less amenable means and without the aid of the cold chain [18,54], thus increasing the potential for the growth of foodborne pathogens in cases where they are introduced into the milk. Kouamé-Sina et al. [57] reported that the average temperature of milk offered for sale in Côte d’Ivoire was 31.9 °C, a temperature that would be optimal for the growth of foodborne pathogens and spoilage microorganisms.

The factors presented in this section show that there is a need to educate milk producers, small-scale processors, and vendors on the importance of refrigerating milk immediately after milking as well as maintaining the cold chain until the milk is heat treated and, subsequently, until the milk is marketed to the consumer. The traditional constraints of poor producer awareness on food safety issues, high capital costs for refrigeration equipment, and lack of awareness of GMPs and GHPs render milk and milk products vulnerable to spoilage and potential risks of foodborne illness.

### 4.2. Chemical Hazards

Milk and milk products can also be contaminated by a variety of chemicals that affect food safety. Chemicals are most often introduced to milk during production through either the ingestion of contaminated animal feedstuffs or the application of veterinary medicines [39,59,60], but they can also be introduced from the environment [61]. Major chemical hazards include mycotoxins, antimicrobial residues, and pesticide residues.

#### 4.2.1. Mycotoxins

Mycotoxins are metabolites produced by fungi or yeast and can be toxic to humans and animals [59]. The main fungal genera that produce mycotoxins include *Alternaria*, *Aspergillus*, *Fusarium*, and *Penicillium*. These fungi commonly contaminate crops used for animal feedstuffs due to a favorable climate and poor pre- and post-harvest practices such as premature or late harvesting of crops, inadequate drying of crops, and storage of crops in high humidity [39]. Multiple mycotoxins can occur simultaneously in the raw ingredients used to make animal feed [62,63]. Once ingested, the mycotoxins are metabolized, biotransformed, and transferred to animal products, such as milk, where they can become a risk to human health if consumed [59].

The main mycotoxins of concern for milk and other dairy products are aflatoxins which are mainly produced by *Aspergillus flavus* and *Aspergillus parasiticus* [61]. Once ingested, aflatoxin B_1_ (AFB_1_) is metabolized into the slightly less toxic aflatoxin M_1_ (AFM_1_) which can appear in milk as early as one day after lactating cows ingest contaminated foodstuffs [64,65]. Aflatoxins are both acutely and chronically toxic and may cause liver cancer, DNA damage, gene mutations, chromosomal anomalies, and cell transformations [61]. Many environmental factors affect the contamination of milk by AFM_1_, but studies show that milk produced during the warm season is less contaminated than milk produced in the cold season most likely due to prolonged storage of feedstuffs in the cold season [66,67]. Additionally, milk produced by animals that are pasture-fed had a lower risk for the presence of aflatoxins [68]. The warm climate and use of small-scale traditional production systems where animals are left to roam the land and graze in West Africa may present a lower risk for aflatoxin contamination.

Other mycotoxins that have been found in milk include fumonisins, zearalenone, ocratoxin, and deoxynivalenol. Fumonisins are produced by fungi from the genus *Fusarium*. The most predominant and most toxic forms include fumonisin B_1_, which is classified as a possible human carcinogen, and fumonisin B_2_ [59,69]. Zearalenone (ZEN) is also produced by the genus *Fusarium* and exhibits estrogenic effects. The estrogenic activity of the metabolite α- zearalanol is greater than that of ZEN [59,70]. Ochratoxin (OTA) is produced mostly by the genera *Aspergillus* and *Penicillium* and has nephrotoxic, hepatotoxic, teratogenic, and immunotoxic activity [59]. Microorganisms in the rumen of cows will degrade OTA, so some studies suggest that there will be minimal excretion in milk [71]. Deoxynivalenol (DON), also known as vomitoxin, is primarily produced by *Fusarium graminearium* and *Fusarium culmorum*. The main product of microbial breakdown in animals is di-epoxy-DON which is less toxic and can be secreted in milk [59]. However, there is limited evidence of this compound occurring naturally in milk ready for human consumption [72].

#### 4.2.2. Antimicrobial Residues

Antibiotics have been used in animal production to control, prevent, and treat infections as well as to promote growth [73]. After entering the animal system through injection or ingestion, most of the antibiotics are metabolized and excreted. However, a portion of the antibiotics (residues) can persist in animal tissues and products such as milk and/or meat [74]. Antibiotics have a maximum residue level (MRL) that is legally allowed in food products obtained from animals receiving veterinary medicine [75]. Food products with residue levels above the MRL can cause a variety of health issues when consumed by humans including the transfer of antibiotic resistance to human pathogenic bacteria, allergic reactions, carcinogenicity, mutagenicity, damage to kidneys or liver, and reproductive disorders [76,77].

Factors that can lead to the presence of antibiotic residues in milk include: failure to comply with withdrawal times (set times after the administration of an antibiotic to a food animal where the residue in a food product is expected to be below the MRL); the irresponsible use of antibiotics in treating diseases in food animals, especially dry cow therapy and mastitis treatment; indiscriminate use of antibiotics as feed additives; and the type of production practiced by the farm (intensive or extensive). Some control strategies for antibiotic residues in milk include (1) the establishment of regulations regarding the proper use of antibiotics in food animals; (2) following proper withdrawal times after the administration of antibiotics; (3) the establishment of proper monitoring systems for the detection of residues using sensitive methods with low rates of false negatives and quantification of residues against the MRL; and (4) education efforts to raise awareness of farmers about antibiotic residues in milk [60,74].

#### 4.2.3. Pesticide Residues

Pesticides are chemical or biological substances intended to repel, destroy, or control pests as well as regulate plant growth [78]. Pesticides may also be applied directly to animals to control skin-dwelling parasites [79]. The proper use of agricultural pesticides can improve agricultural productivity, protect crop losses, and increase the availability of quality food [80]. However, uncontrolled pesticide application or improper disposal can leave residues that may persist for extended times in the environment and may ultimately cause adverse health effects in humans such as allergies, asthma, immune suppression, hormone disruption, neurological diseases, reproductive abnormalities, and cancer [80,81,82].

Pesticide use in Africa is mainly for large-scale farming, especially cash crops. However, studies have shown that poor handling of these pesticides can occur, including incorrect dosage and application, as well as leakage from storage containers. Pesticides classified as organochlorines are of main concern. Examples of these include insecticides, such as aldrin, dieldrin, and dichlorodiphenyltrichloroethane (DDT); fungicides, such as hexachlorobenzene (HCB); and industrial chemicals, such as polychlorinated biphenyls (PCBs). The ubiquitous nature, long-term environmental persistence, and lipophilic properties of organochlorine pesticides mean that they can accumulate in animal-based food products such as milk, and they may be found in even greater concentrations in milk-based products such as butter or cheese [82,83,84].

Although numerous organochlorine pesticides have been banned for use in agriculture around the world, they have continued to be used in many African countries. For example, DDT is still used through an exemption of approved disease vector control [84]. Similar to antimicrobials, pesticides have a maximum residue level (MRL) for food products. Many African countries have adopted pesticide MRLs from the Codex Alimentarius or the importing country, but some countries, such as Nigeria and Ghana, have also developed their own regulatory authorities [79].

### 4.3. Physical Hazards

Physical hazards consist of foreign materials or objects not naturally present in food products and can include sharp hazards; choking hazards; animal food hazards, such as size and hardness; or filth, such as dirt, feces, and insect parts. Physical hazards can cause injuries, such as damage to the oral cavity or gastrointestinal tract, choking, or microbiological contamination [85,86]. Contamination of milk by physical hazards occurs most often on the farm or during processing [87]. On the farm, physical hazards may arise from soil, feed, bedding, feces, or hair that can adhere to the exterior surface of the udder and pollute the milk during collection [48]. Physical hazards may also be introduced to milk by uncleaned hands or collection/storage equipment. The cleaning of teats, hands, and milking equipment before milk collection can reduce the physical contaminants in the milk [36]. The introduction of physical hazards during processing can also occur. This may be due to equipment (e.g., metal parts or rubber shreds), personnel (e.g., jewelry), or raw materials (e.g., leftover from the farm) [87]. Good Agricultural Practices (GAPs), Good Manufacturing Practices (GMPs), and Hazard Analysis Critical Control Points (HACCP) systems, such as visual inspection and metal detection, can all help prevent the contamination of milk by physical hazards [86,87].

## 5. Processing Technologies for Milk and Milk Products

While the demand for milk in West Africa is increasing, milk production has been growing at a very slow rate. Africa’s warm climate makes the preservation of highly perishable foods, such as milk, difficult, and the lack of processing or preservation resources, such as refrigeration, in some communities exacerbates this [58]. The production of milk or milk products in Africa traditionally has very few steps from the production of raw milk to the sale of milk and milk products to consumers [36]. These steps may or may not include milk-processing steps. Food-processing methods are meant to modify food ingredients and raw materials to produce safe foods with desired quality attributes [58].

### 5.1. Cooling Practices

The rapid cooling of raw milk after collection is important to prevent the growth of microorganisms before further processing treatments. Different countries have varying regulations on what temperature raw milk should be cooled to before processing that range from 4 to 8 °C [36,55,56,88]. Some in the dairy industry will store raw milk at even lower temperatures to minimize the growth of psychrotrophic microorganisms which are able to grow at refrigeration temperatures [89]. However, the guidance document “Guide to Good Hygiene Practices: Quality Control in Dairy Processing in Senegal” does not mention the need for the cooling of raw milk. Instead, the document states that milk should be transported to the collection center or processor within 3 h after milking [16]. The absence of guidance for cooling may be due to the lack of availability of cooling facilities for small-scale producers and processors due to the high initial cost of investing in these facilities, the high cost to run these facilities, and the lack of reliable electricity [36]. In fact, Chengat Prakashbabu et al. [35] found that only about 3% of milk producers in Senegal regularly chilled their milk after collection.

### 5.2. Heat Treatments

The proper heat treatment of raw milk can significantly increase the safety of milk and milk products. The main goals for the heat treatment of milk are to reduce or eliminate both pathogenic and spoilage microorganisms, to inactivate enzymes, and to minimize chemical reactions and physical changes that may affect quality [90]. There are three general categories of heat treatment based on the time and temperature combinations applied. The first is thermization which heats milk to a temperature between 57 and 68 °C for 15 to 20 s [90]. Thermization leads to approximately a 3 to 4 log reduction of vegetative bacteria which means that not all microorganisms present in the milk will be destroyed [91]. The second heat treatment type is pasteurization where the milk is heated to either 63°C for 30 min or 72 °C for 15 s with some variations based on the country [90]. Pasteurization leads to the elimination of all vegetative microorganisms pathogenic to humans, but it is not able to destroy some resistant vegetative microorganisms, preformed heat-resistant enterotoxins, or heat-resistant spores [91]. The final heat treatment type for raw milk is sterilization or ultra-high temperature processing. Sterilization is achieved at temperatures between 110 and 120 °C for 10 to 20 min while in ultra-high temperature processing, milk is heated to between 135 and 150 °C for 2 to 10 s [90,91]. These treatments destroy vegetative microorganisms and most spores with a minimum of a 12-log reduction, as well as destroying most toxins, except for the emetic toxin of *B. cereus* which is very heat resistant. Ultra-high temperature is preferred over sterilization because the shorter processing times result in less loss of quality [91]. Additionally, none of the heat treatments are likely to influence aflatoxin levels that may be present in the milk [61].

Boiling or heating raw milk is sometimes practiced in Africa because of the lack of refrigeration, and the milk may be heated several times as a means of preservation. However, the time and temperature combinations used are rarely controlled and may not sufficiently reduce or eliminate microorganisms depending on the initial levels of contamination or the type of microorganism present (e.g., spore-forming bacteria) [36]. Published studies from West Africa show that boiling milk is quite rare, and even if individuals were aware that boiling milk could prevent some milk-borne diseases, they were not doing it because of tradition, superstition, lack of familiarity with the process, or lack of equipment necessary to boil milk [18,35,54,57].

### 5.3. Fermentation

Fermentation is one of the oldest methods of food processing, and many traditional techniques for fermentation have been handed down through generations. Fermentation serves as an important form of preservation in Africa because it is a relatively cheap and convenient method of prolonging the shelf-life, increasing diversity, and improving the digestibility and nutritional value of milk and milk products [36,58].

During fermentation, desirable changes in a food product occur due to the metabolic activity of microorganisms which create the characteristic flavor, texture, and color of fermented food products [34,92]. Lactic acid bacteria (LAB), including *Lactobacillus*, *Lactococcus*, *Weissella*, *Leuconostoc, Streptococcus,* and *Enterococcus*, are the dominant microorganisms driving the fermentation of milk in Africa [93,94]. LAB metabolizes sugars and produces a variety of organic acids and other antimicrobial compounds, such as ethanol, bacteriocins, and hydrogen peroxide, during fermentation. The production of organic acids causes a decrease in the pH of the milk which leads to suppression of the growth and survival of spoilage and pathogenic microorganisms that may be present in the milk [34,92,95].

The antimicrobial effects of LAB mean that fermented foods are generally considered safe. However, most traditional techniques used for African fermented dairy products have the potential to lead to variations in the quality of the final products. Most African fermented dairy products use spontaneous fermentation where fermentation is achieved from microorganisms naturally present in the raw materials or from the environment instead of from well-defined starter cultures. Additionally, back-slopping may be used where a part of a previous batch of fermented food is used to start the next batch. Finally, temperatures and durations of fermentation are not well controlled [34,36,58].

## 6. Safety and Quality of Milk and Milk Products

The predominance of informal dairy sectors in West Africa, which produce mostly raw, unpasteurized milk or traditional, spontaneously fermented milk products that are sold through small-scale networks without a cold chain, increases the risk of food safety hazards [18,35,39]. In addition, the contamination of many milk products in West African countries arises from a lack of GHPs and GMPs including hand washing, udder cleaning, and storage and transportation conditions as well as the use of improper containers and milking of cows undergoing antibiotic treatments [54]. Table 1 and Table 2 summarize some recent literature on the microbiologic and chemical quality, respectively, of milk and milk products in West Africa. According to Breurec et al. [44], efforts should be made to implement good hygiene practices during milk collection and processing including pasteurization, transportation, and distribution. Furthermore, an inspection of milk production and helping farmers with fodder quality could be important steps to improve milk production and processing in Senegal.

### 6.1. Microbiological Quality

In the literature presented in Table 1, cows were the most common milk source with only one study each presenting data on camel or goat milk or reconstituted milk powder. Most studies presented data on raw milk (*n* = 16) with others presenting data on some form of traditional fermented milk product, such as *gappal*, *lait caillé*, *nunu*/*nono*, or *brukina*. Additionally, two studies presented data on non-fermented traditional cheeses (*wagashi* and *wara*). Only five studies presented results for boiled or pasteurized milk. These studies represent the market for the vast majority of milk and milk products sold in West Africa because of the developing dairy networks and the lack of a cold chain infrastructure in most of these countries.

The most studied microorganism in the literature was *E. coli* (*n* = 16). *E. coli,* along with coliform bacteria (*n* = 7), can be used as indicators of sanitary quality in food products, including milk. These microorganisms do not necessarily indicate the presence of pathogens but provide a quantitative measure of microbial contamination in food products [129]. Many countries have established limits for indicator microorganisms in dairy products. For example, in the United States, raw milk should not exceed 5 log CFU/mL bacteria levels, and coliform levels for Grade “A” pasteurized milk are not to exceed 1 log CFU/mL [55]. In the European Union, raw cow’s milk should not exceed 5 log CFU/mL for plate count bacteria while pasteurized milk and pasteurized liquid milk products should not exceed 5 CFU/mL of Enterobacteriaceae [56]. The studies presented here reported coliform levels ranging from 0.72 to 7.51 log CFU/mL for all products. The major pathogens reported in the literature included *S. aureus* (*n* = 9), *Salmonella* spp. (*n* = 5), *E. coli* O157:H7 (*n* = 2), and *L. monocytogenes* (*n* = 2). These pathogens are among the leading causes of foodborne disease worldwide [1].

### 6.2. Chemical Quality

The literature presented in Table 2 reported data on similar products as the microbiological quality articles. Most reported data on raw milk samples with other traditional milk products, such as *wagashi*, *nono*, *kindirmo*, and *wara*, also being tested. Five studies reported data on the presence of the mycotoxin aflatoxin M_1_ with prevalence rates ranging from 10% to 100%. One reason for the focus on mycotoxins in West Africa could be the availability of analytical methods tailored to detect aflatoxin M_1_ [39]. Recommended maximum aflatoxin levels in food products, including milk, range from 0.5 to 15 µg/kg according to the World Health Organization [130]. There are a number of other mycotoxins that could potentially contaminate dairy products in West Africa, so more research and development of accessible analytical methods are needed.

Studies on the presence of antimicrobial residues in milk focused mainly on raw milk with only one study sampling the traditional products *wara* and *nono*. Similar to mycotoxins, the range of prevalence varied widely from 3.1% to 100%. The consumption of antimicrobial residues in food products could lead to the development of multidrug-resistant strains of pathogens in humans. Additionally, the antimicrobial residues themselves can lead to adverse health effects such as allergic reactions, carcinogenicity, mutagenicity, damage to kidneys or liver, and reproductive disorders [76,77].

Only one recently published study on the presence of pesticide residues in animal milk in West Africa was found. All samples tested from raw milk, yogurt, and cheese had pesticide residues present with a prevalence ranging from 61% to 95%. The ubiquitous nature, long-term environmental persistence, and lipophilic properties of organochlorine pesticides specifically mean that they can accumulate in animal-based food products, such as milk, and they may be found in even greater concentrations in milk-based products, such as cheese [82,83,84]. This seems to be supported by Darko and Acquaah [128] where organochlorine pesticide residue concentrations for milk and yogurt ranged from 0.22 μg/kg for Aldrin to 12.53 μg/kg for *p,p’*-DDT and 0.02 μg/kg for Dieldrin to 7.38 μg/kg for *p,p’*-DDT, respectively, while concentrations in cheese ranged from 2.36 μg/kg for Aldrin to 118.25 μg/kg for *p,p’*-DDT. High levels of these pesticide residues can lead to adverse health effects in humans such as allergies, asthma, immune suppression, hormone disruption, neurological diseases, reproductive abnormalities, and cancer [80,81,82].

## 7. Conclusions

Consumers in Senegal have an increasing preference for local and domestically produced and processed milk compared to imported milk. However, imported milk and milk products are more accessible, diverse, and safe, which influences consumers’ decisions when purchasing. Efforts have been made to improve local dairy production by establishing large, organized dairies that collect milk from rural production areas and developing small-scale processing units such as mini dairies. However, there is a need to educate milk producers, small-scale processors, and vendors on common food safety measures for milk and milk products as well as assist them in obtaining the equipment necessary to keep products safe.

## Figures and Tables

**Table 1:** Microbiological quality of milk and milk products in West Africa ^1^.

Country	Milk Source	Milk Product	Processing Technique	Microorganism	Prevalence n/N ^2^ (%)	Mean log CFU ^3^/mL	Reference
Benin	Cow	Raw milk	None	Fecal coliform bacteria	NR ^4^	2.96	[96]
				*Escherichia coli*		0.60	
				*Staphylococcus aureus*		1.60	
Benin	Cow	Raw milk	None	*Listeria monocytogenes*	9/30 (30.0)	NR	[97]
Burkina Faso	NR	Raw milk	None	*E. coli*	19/50 (38.0)	NR	[98]
				*Salmonella enterica*	3/50 (6.0)		
		Curd milk	NR	*E. coli*	22/50 (44.0)		
Burkina Faso	NR	Raw milk	None	*E. coli*	5/15 (33.0)	NR	[45]
				*Klebsiella* spp.	15/15 (100.0)		
				*K. pneumoniae*	10/15 (66.7)		
				*Enterobacter* spp.	13/15 (86.7)		
				*Enterobacter cloacae*	10/15 (66.7)		
		Sour milk	NR	*E. coli*	2/15 (13.3)		
				*Klebsiella* spp.	5/15 (33.3)		
				*K. pneumoniae*	3/15 (20.0)		
				*Enterobacter* spp.	3/15 (20.0)		
				*Enterobacter cloacae*	1/15 (6.7)		
		Yogurt	NR	*E. coli*	1/15 (6.7)		
				*Klebsiella* spp.	4/15 (26.7)		
				*K. pneumoniae*	4/15 (26.7)		
				*Enterobacter* spp.	2/15 (13.3)		
				*Enterobacter cloacae*	2/15 (13.3)		
Burkina Faso	Cow	*Gappal*	Combined fermentation of raw or sour milk and millet dough	Enterobacteriaceae	13/106 (12.3)		[99]
				*S. aureus*	10/106 (9.4)		
Burkina Faso	Cow	Farm milk	None	*E. coli*	68/69 (98.6)	NR	[100]
		Raw milk	None		29/84 (34.5)		
		Curd milk	NR		29/89 (32.6)		
		Pasteurized milk	NR		29/101 (28.7)		
		Yogurt	NR		4/92 (4.4)		
		*Déguè*	NR		14/87 (16.1)		
Burkina Faso	Camel	Fermented milk	NR	Coliform bacteria	21/24 (87.5)	NR	[101]
				*S. aureus*	19/24 (79.2)		
	Cow			Coliform bacteria	45/50 (90.0)		
				*S. aureus*	42/50 (84.0)		
	Goat			Coliform bacteria	33/40 (82.5)		
				*S. aureus*	31/40 (77.5)		
Burkina Faso	Cow	*Lait caillé*	Spontaneous fermentation in covered plastic containers at ambient temperature	Enterobacteriaceae	NR	5.80	[102]
				*Enterococcus faecium*		NR	
Côte d’Ivoire	Reconstituted powdered milk	*Lait caillé*	Fermentation started with addition of commercial yogurt	Coliform bacteria	99/100 (99.0)	4.63	[103]
				*E. coli*	51/100 (51.0)	4.08	
				*Salmonella* spp.	57/100 (57.0)	NR	
Côte d’Ivoire	Cow	Raw milk	None	*E. coli*	NR/NR (70.5)	NR	[104]
				*S. aureus*	NR/NR (17.6)		
				*Enterococcus* spp.	NR/NR (58.8)		
Ghana	Cow	*Nunu*	Spontaneous fermentation in calabashes or rubber buckets for 1–2 days	*Enterobacter*	NR	NR	[105]
				*Klebsiella*			
				*E. coli*			
				*Proteus vulgaris*			
				*Shigella*			
Ghana	Cow	Raw milk	None	*Yersinia* spp.	19/98 (19.9)	NR	[42]
				*Klebsiella* spp.	15/98 (15.6)		
				*Proteus* spp.	7/98 (7.3)		
				*Enterobacter* spp.	6/98 (6.3)		
				*E. coli*	2/98 (2.1)		
				*Staphylococcus* spp.	14/98 (14.6)		
				*Bacillus* spp.	11/98 (11.5)		
				*Mycobacterium* spp.	1/98 (1.0)		
Ghana	Cow	Raw milk	None	*E. coli*	28/224 (12.5)	NR	[106]
Ghana	Cow	*Nunu*	Spontaneous fermentation in calabashes or rubber buckets for 1–2 days	*Enterococcus faecium*	NR	NR	[32]
Ghana	Cow	Raw milk	None	*E. coli*	74/150 (49.3)	NR	[107]
Ghana	Cow	*Brukina*	Milk was boiled for 1 h then fermentation was started by back slopping and continued overnight at room temperature	Coliform bacteria	NR	2.91	[108]
				*S. aureus*		4.68	
Ghana	Cow	Raw milk	None	*Listeria* spp.	20/114 (17.5)	NR	[109]
				*L. monocytogenes*	10/114 (8.8)		
		*Nunu*	Spontaneous fermentation	*Listeria* spp.	11/84 (13.1)		
				*L. monocytogenes*	4/84 (4.7)		
Ghana	Cow	Raw milk	None	*E. coli* O157:H7	1/58 (1.7)	NR	[110]
		Boiled milk	Boiling	*E. coli* O157:H7	12/19 (63.2)		
				*S. aureus*	6/19 (31.6)		
		*Nunu*	Spontaneous fermentation	*E. coli* O157:H7	3/9 (33.3)		
				*S. aureus*	1/9 (11.1)		
		*Brukina*	Fermented milk and millet	*E. coli* O157:H7	6/21 (28.6)		
				*S. aureus*	1/21 (4.8)		
		Raw *wagashi*	Soft cheese made without fermentation	*E. coli* O157:H7	1/17 (5.9)		
				*Salmonella enterica*	3/17 (17.7)		
Ghana	Cow	Boiled milk	Boiling over open flame for up to 1 h	Fecal coliform bacteria	NR	0.72	[111]
				*E. coli*		NR	
				*K. pneumoniae*		NR	
		*Brukina*	Fermented milk and millet	Fecal coliform bacteria		2.59	
				*E. coli*		NR	
				*K. pneumoniae*		NR	
		Raw *wagashie*	Coagulating fresh milk with extracts of the Sodom apple plant	Fecal coliform bacteria		4.97	
				*E. coli*		NR	
				*K. pneumoniae*		NR	
		Fried *wagashie*	Deep frying	*E. coli*		NR	
				*K. pneumoniae*		NR	
		Yogurt	NR	Fecal coliform bacteria		1.43	
				*E. coli*		NR	
				*K. pneumoniae*		NR	
Mali	Cow	Raw milk	None	Coliform bacteria	5/6 (83.3)	4.21 ^5^	[112]
				*E. coli*	2/6 (33.3)	5.99 ^5^	
		Curdled milk	NR	Coliform bacteria	4/12 (33.3)	6.76 ^5^	
				*E. coli*	4/12 (33.3)	6.66 ^5^	
Nigeria	NR	Pasteurized milk	NR	*Salmonella* spp.	2/13 (15.4)	1.83	[113]
				*Streptococcus* spp.	5/13 (38.5)	3.32	
				*Enterococcus* spp.	3/13 (23.1)	3.34	
				*Pseudomonas* spp.	3/13 (23.1)	2.38	
		Yogurt		*E. coli*	4/15 (26.7)	1.48	
				*S. aureus*	3/15 (20.0)	3.37	
				*Streptococcus* spp.	9/15 (60.0)	3.48	
				*Enterococcus* spp.	3/15 (20.0)	3.15	
				*Klebsiella* spp.	2/15 (13.3)	3.22	
Nigeria	NR	Raw milk	None	*S. aureus*	6/80 (7.5)	NR	[114]
		*Nono*	Fermentation		8/80 (10.0)		
Nigeria	Cow	Raw milk	None	*S. aureus*	17/100 (17.0)	NR	[115]
				MRSA ^6^	15/100 (15.0)		
		*Wara*	NR	*S. aureus*	35/100 (35.0)		
				MRSA	35/100 (35.0)		
Nigeria	Cow	Raw milk	None	*E. coli*	15/160 (9.4)	NR	[116]
				*E. coli* O157:H7	3/160 (1.9)		
		Fermented milk	NR	*E. coli*	9/100 (9.0)		
				*E. coli* O157:H7	2/100 (2.0)		
Nigeria	Cow	*Nono*	Fermentation	*E. coli*	27/100 (27.0)	NR	[117]
				*Enterobacter* spp.	8/100 (8.0)		
				*Klebsiella* spp.	6/100 (6.0)		
				*Proteus* spp.	3/100 (3.0)		
				*Citrobacter* spp.	2/100 (2.0)		
Senegal	NR	Raw milk	None	Coliform bacteria	NR	4.93	[44]
				*Salmonella* Johannesburg	1/15 (6.7)	NR	
				*Coxiella burnetii*	6/15 (40.0)	NR	
				Coagulase-positive *Staphylococcus*	NR	NR	
		Pasteurized milk	NR	Coliform bacteria	NR	7.51	
				Coagulase-positive *Staphylococcus*	NR	NR	

^1^ Adapted from [39]. ^2^ Number of positive samples/total number of samples. ^3^ Colony forming units. ^4^ Not reported. ^5^ Most probable number (MPN)/100 mL. ^6^ Methicillin-resistant *S. aureus.*

**Table 2:** Chemical quality of milk and milk products in West Africa ^1^.

Country	Milk Product	Chemical Hazard	Concentration	Prevalence n/N ^2^ (%)	Reference
**Mycotoxins**					
Benin	*Wagashi* cheese	Aflatoxin M1	Not detected	Not detected	[118]
Nigeria	Powdered milk	Aflatoxin M1	0.136 µg/kg	19/83 (22.9)	[119]
Nigeria	Raw cow milk	Aflatoxin M1	0.665 µg/L	10/10 (100.0)	[120]
	*Nono*		0.924 µg/L	10/10 (100.0)	
	*Kindirmo*		0.575 µg/L	10/10 (100.0)	
Nigeria	Raw cow milk	Aflatoxin M1	3000–7000 ng/L	NR ^3^	[121]
	*Wara* cheese				
Nigeria	Raw cow milk (Nomadic)	Aflatoxin M1	0.531 µg/L	16/20 (80.0)	[122]
	Raw cow milk (Commercial)		0.058 µg/L	5/20 (25.0)	
	*Nono*		0.592 µg/L	7/20 (35.0)	
	Yogurt		0.615 µg/L	2/20 (10.0)	
	Cheese		0.588 µg/L	8/20 (40.0)	
**Antimicrobial Residues**					
Ghana	Raw cow milk	Antibiotic residues	NR	7/224 (3.1)	[106]
Mali	Raw milk	Antibiotic residues	NR	14/220 (6)	[123]
Niger	Raw milk	Antibiotic residues	NR	19/192 (9.9)	[124]
Nigeria	Raw Goat Milk (red Sokoto breed)	Penicillin	0.282 ppm	100/100 (100)	[125]
		Amoxicillin	0.123 ppm	100/100 (100)	
	Raw Goat Milk (West African dwarf breed)	Penicillin	0.257 ppm	66/66 (100)	
		Amoxicillin	0.108 ppm	66/66 (100)	
Nigeria	Raw cow milk	Penicillin G	15.22 µg/L	135/328 (41.1)	[126]
	*Wara* cheese		8.24 µg/L	73/180 (40.2)	
	*Nono* fermented milk		7.60 µg/L	22/90 (24.4)	
Senegal	Raw cow milk	Chloramphenicol	NR	32/41 (78.0)	[127]
**Pesticide Residues**					
Ghana	Raw milk	Lindane	<LD ^4^	13/20 (66.0)	[128]
		Aldrin	0.22 μg/kg	12/20 (61.0)	
		Endosulfan	0.60 μg/kg	14/20 (72.0)	
		*p*,*p′*-DDE ^5^	1.42 μg/kg	16/20 (82.0)	
		Dieldrin	1.32 μg/kg	17/20 (83.0)	
		*p*,*p′*-DDT ^6^	12.53 μg/kg	15/20 (75.0)	
	Yogurt	Lindane	0.02 μg/kg	41/60 (68.0)	
		Aldrin	0.07 μg/kg	43/60 (72.0)	
		Endosulfan	0.06 μg/kg	44/60 (74.0)	
		*p*,*p′*-DDE	0.88 μg/kg	55/60 (91.0)	
		Dieldrin	0.11 μg/kg	43/60 (72.0)	
		*p*,*p′*-DDT	7.38 μg/kg	57/60 (95.0)	
	Cheese	Lindane	<LD–4.41 μg/kg	51/60 (85.0)	
		Aldrin	2.36 μg/kg	44/60 (73.0)	
		Endosulfan	3.42 μg/kg	37/60 (62.0)	
		*p*,*p′*-DDE	106.91 μg/kg	56/60 (94.0)	
		Dieldrin	7.42 μg/kg	43/60 (71.0)	
		*p*,*p′*-DDT	118.25 μg/kg	57/60 (95.0)	

^1^ Adapted from [39]. ^2^ Number of positive samples/total number of samples. ^3^ Not reported. ^4^ Limit of detection. ^5^ Dichlorodiphenyldichloroethylene. ^6^ Dichlorodiphenyltrichloroethane.

## Data Availability

No new data were created or analyzed in this study. Data sharing is not applicable to this article.
